# Low endemism, continued deep-shallow interchanges, and evidence for cosmopolitan distributions in free-living marine nematodes (order Enoplida)

**DOI:** 10.1186/1471-2148-10-389

**Published:** 2010-12-18

**Authors:** Holly M Bik, W Kelley Thomas, David H Lunt, P John D Lambshead

**Affiliations:** 1 Nematode Research Group, Department of Zoology, The Natural History Museum, Cromwell Road, London SW7 5BD, UK; 2 Hubbard Center for Genome Studies, University of New Hampshire, 35 Colovos Road, Durham, NH 03824, USA; 3 Department of Biological Sciences, University of Hull, Cottingham Road, Hull HU6 7RX, UK; 4 School of Ocean and Earth Science, National Oceanography Centre, European Way, Southampton SO14 3ZH, UK

## Abstract

**Background:**

Nematodes represent the most abundant benthic metazoa in one of the largest habitats on earth, the deep sea. Characterizing major patterns of biodiversity within this dominant group is a critical step towards understanding evolutionary patterns across this vast ecosystem. The present study has aimed to place deep-sea nematode species into a phylogenetic framework, investigate relationships between shallow water and deep-sea taxa, and elucidate phylogeographic patterns amongst the deep-sea fauna.

**Results:**

Molecular data (18 S and 28 S rRNA) confirms a high diversity amongst deep-sea Enoplids. There is no evidence for endemic deep-sea lineages in Maximum Likelihood or Bayesian phylogenies, and Enoplids do not cluster according to depth or geographic location. Tree topologies suggest frequent interchanges between deep-sea and shallow water habitats, as well as a mixture of early radiations and more recently derived lineages amongst deep-sea taxa. This study also provides convincing evidence of cosmopolitan marine species, recovering a subset of Oncholaimid nematodes with identical gene sequences (18 S, 28 S and *cox1*) at trans-Atlantic sample sites.

**Conclusions:**

The complex clade structures recovered within the Enoplida support a high global species richness for marine nematodes, with phylogeographic patterns suggesting the existence of closely related, globally distributed species complexes in the deep sea. True cosmopolitan species may additionally exist within this group, potentially driven by specific life history traits of Enoplids. Although this investigation aimed to intensively sample nematodes from the order Enoplida, specimens were only identified down to genus (at best) and our sampling regime focused on an infinitesimal small fraction of the deep-sea floor. Future nematode studies should incorporate an extended sample set covering a wide depth range (shelf, bathyal, and abyssal sites), utilize additional genetic loci (e.g. mtDNA) that are informative at the species level, and apply high-throughput sequencing methods to fully assay community diversity. Finally, further molecular studies are needed to determine whether phylogeographic patterns observed in Enoplids are common across other ubiquitous marine groups (e.g. Chromadorida, Monhysterida).

## Background

The marine environment encompasses nearly 71% of the earth's surface, with deep-sea habitats (> 400 m depth) representing 91% of the total bottom surface area of the world ocean [1]. Given the vastness of this ecosystem, it is likely that deep-sea fauna constitute a substantial proportion of the Earth's biodiversity; understanding the diversity, abundance, and ecological roles of deep-sea taxa is crucial for quantifying biological contributions to global nutrient cycles such as carbon flux [2]. Assessing the scale of this role is largely dependant on accurately characterizing the abundance and diversity of deep-sea biota, as decreasing biodiversity has been linked to exponential reductions in ecosystem functioning [3].

Nematodes represent the most abundant metazoan phylum in deep-sea sediments, comprising 85-96% of all benthic meiofauna [4]. We lack an accurate characterization of species diversity and phylogeographic patterns in this dominant benthic group--a situation hindered by logistical difficulties in sampling the deep-sea and a persistent shortage of nematode taxonomists. Currently, we do not understand what proportion of the deep-sea nematode fauna represents unique taxa (novel species, endemic lineages) and what fraction instead represents a subset of shallow water biodiversity. Fossil evidence [5,6] proposes that deep-sea species originally represented invasions from nearby shallow-water habitats, but 'endemic' lineages developed over time as taxa retreated from shallow water habitats altogether and became strictly deep-sea groups. Evidence from isopods suggests that deep-sea taxa represent a mix of older endemic lineages and fauna derived more recently from shallow water forms. Within the Janiroidea, there is evidence supporting the radiation of shallow-water isopods into deeper habitats through isothermal water columns [7], reinvasions of shallow habitats by deep-sea taxa [7,8], and multiple independent colonizations of the deep-sea [9]--thus, deep-sea taxa within this group probably represent ancient evolutionary lineages that split off early from other taxa [10]. On the other hand, the Flabellifera contains no endemic deep-sea isopod families (in contrast to the Janiroidea which contains seven), and exhibits low diversity in deep habitats [10]; this group is thought to have arisen relatively recently, with shallow-water taxa invading deep-sea habitats subsequent to anoxic oceanic conditions that prompted mass deep-water extinctions in the Palaeocene.

It is unknown whether extant deep-sea nematode species represent relatively recent radiations or older lineages. For small immobile animals such as nematodes, historical expansions and contractions of anoxic zones may have played a large role in shaping phylogeographic patterns amongst taxa [11]. Although nematodes are able to survive low oxygen conditions and short-term anoxia [12,13], they appear unable to survive in permanently anoxic sediments [14]. The scale of past anoxic events is largely unknown, and it is possible that some oxygen minimum zones were patchy and localized rather than globally encompassing the entire deep-sea [11,15]; thus, different depths (e.g. abyssal versus bathyal) may have acted variably as source or sink habitats at different timepoints. Many deep-sea genera are cosmopolitan in their occurrence [1], but there is little information on species distributions within this habitat. Limited data from shallow water nematodes indicates that some nematode species have large ranges [16,17], but it is unknown whether or not deep-sea taxa possess similar dispersal capabilities.

Our investigation of deep-sea nematodes has focused on the order Enoplida, a diverse group of species (encompassing many predacious taxa) commonly found in marine sediments. Using up to three gene sequences from individual specimens (18 S rRNA, 28 S rRNA, and *cox1*), our narrow taxonomic focus has allowed for an intensive assessment of evolutionary relationships between shallow-water and deep-sea taxa representing multiple ocean basins and a range of collection depths. More concentrated sampling within certain genera has allowed further assessment of geographic patterns within specific deep-sea lineages. By clarifying the phylogenetic placement of deep-sea species, our investigation represents another important step towards a comprehensive molecular framework of the Nematoda. In addition, this study is elucidating biogeographic and bathymetric patterns for the first time using molecular data from deep-sea nematode species; characterizing genetic diversity within this extensive habitat is crucial for quantifying global nematode diversity.

## Results

**Figure 1:**
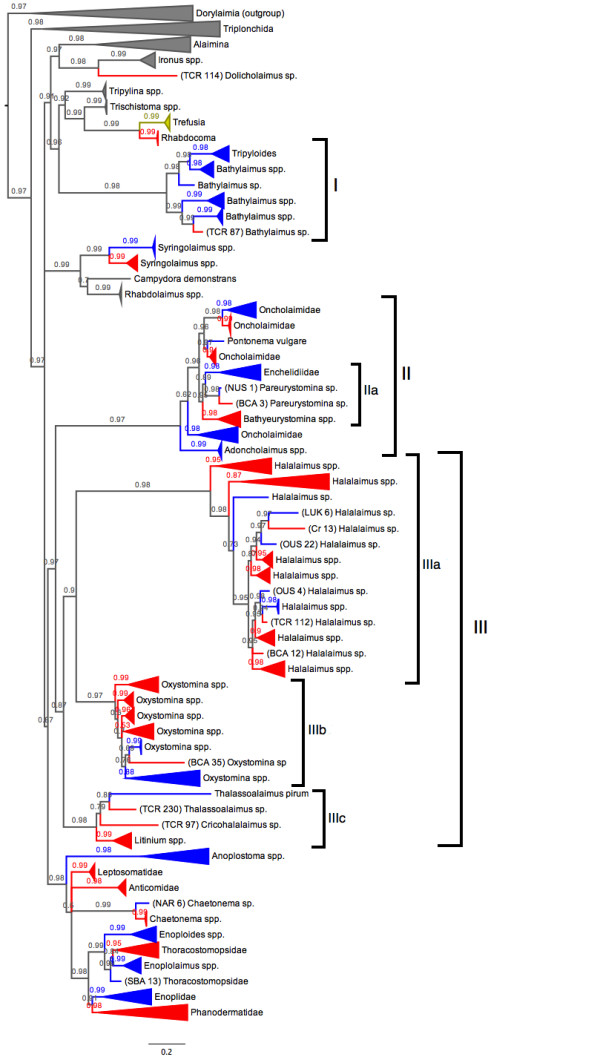
**Bayesian phylogeny based on SSU data displaying the habitats of marine nematodes within the Enoplida**. Red clades indicate deep-sea species, blue clades represent shallow water species, and yellow clades contain species from both environments.

**Figure 2:**
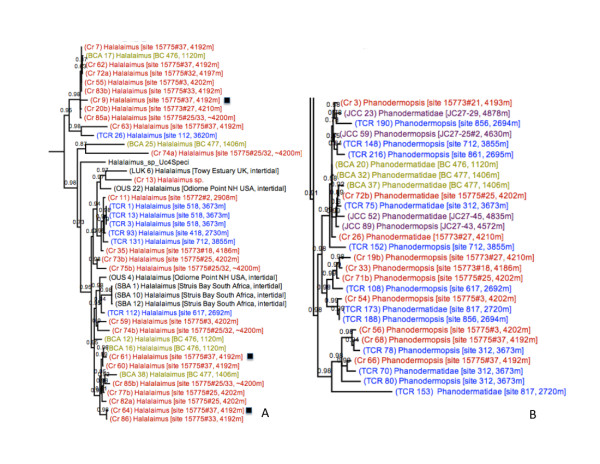
**Bayesian phylogeny built using SSU sequences, showing the collection depths of deep-sea specimens within the genus *Halalaimus *(A) and family Phanodermatidae (B)**. Colors equate to collection location of samples: Black = intertidal, Blue = deep-sea Pacific, Red = deep-sea Southern Indian Ocean, Yellow = Antarctic shelf.

SSU tree topologies provide substantial insight regarding the evolution of deep-sea species. Phylogenetic relationships amongst Enoplid taxa were overwhelmingly congruent in both Maximum Likelihood and Bayesian tree topologies. Deep-sea nematodes represent a wide taxonomic and phylogenetic breadth within the Enoplida. Deep-sea species were recovered in most marine families--the absence of representatives within some clades may be due to limited sampling. Within genera, phylogenies recovered deep-sea species and shallow water species as sister taxa, as clearly seen for *Syringolaimus*, *Bathylaimus*, *Oxystomina*, *Halalaimus*, and *Chaetonema *species (Figure 1); deep-sea taxa do not form an independent lineage within the Enoplida. Within more intensively sampled clades (Figure 2), there does not appear to be any observed clustering pattern according to sample depth or geographic location.

High pairwise sequence identities were observed between many deep-sea and shallow water species, implying a recent divergence between taxa in these habitats. Phylogenetic structures further suggest both frequent and recent interchanges between habitats--there appear to be multiple, independent evolutionary origins for both shallow water and deep-sea taxa. Within the well-sampled genus *Halalaimus *(Clade IIIa, Figure 1), shallow water lineages appear to have arisen independently at least three times from deep-sea lineages, and there is further evidence for at least three independent invasions of the deep sea. Extant shallow water lineages in Clades IIIb and IIIc (Figure 1) also seem to represent additional derivations from deep-water taxa. Habitat transitions in the opposite direction (shallow to deep) are instead implied by the structure outlined in Clades I and II (Figure 1); here, deep-sea taxa are recovered as recent splits within primarily shallow clades. The presence of highly divergent, early branching deep-sea lineages within some clades (Clades IIa, IIIa and IIIb, Figure 1) also suggests a more ancient origin for some groups of deep-sea nematodes.

Sequence data from multiple genes provides some evidence for widespread (and potentially cosmopolitan) distributions in a small fraction of nematode species. Pairwise sequence identity comparisons within SSU, LSU, and *cox1 *datasets revealed a subset of specimens possessing identical gene sequences. Identical gene sequences were, unsurprisingly, most often observed amongst specimens collected at the same sample site. However, a number of specimens shared identical 18 S and 28 S ribosomal sequences (and additionally *cox1 *haplotypes) despite being collected from disparate geographic locations (Additional file 1, Table S1). The highest number of identical gene copies were recovered from nematodes belonging to the Oncholaimidae (genera *Oncholaimus *and *Viscosia*) collected from intertidal sediments; the distance between sample sites varied from tens to thousands of kilometres (Additional file 1, Table S1). One set of *Viscosia *specimens were isolated from Devon, England, the Clyde Estuary in Scotland and New Hampshire, USA. Another set of *Oncholaimus *specimens were collected from at two sites in Massachusetts, USA as well as at two South African beaches. Where available, analysis of mitochondrial sequences showed that nematodes within these two groups additionally shared mitochondrial haplotypes. One group of *Oncholaimus *specimens with identical ribosomal sequences exhibited two primary mt haplotypes (denoted as B and C in Additional file 1, Table S1); these haplotype sequences only differed by 2 substitutions. Little mitochondrial data was obtained from deep-sea nematodes (due to a low success rate of *cox1 *primers), but identical ribosomal sequences were isolated from the Antarctic shelf (*Syringolaimus *and *Oxystomina*, Additional file 1, Table S1), bathyal sediments (Thoracostomopsidae and Phanodermatidae, Additional file 1, Table S1), and abyssal habitats (*Halalaimus *and *Chaetonema*, Additional file 1, Table S1). Our data further recovered identical gene sequences from *Trefusia *specimens in the Clyde estuary and Antarctic shelf (Additional file 1, Table S1). Unfortunately, only one specimen was recovered from the Antarcitic shelf site, but this result was surprising given the large distance (~15,000 km) and depth gradient (670 m) between the two sites.

## Discussion

### No endemic deep-sea lineages and frequent deep/shallow interchanges

Prior to this investigation, there were no public data available for reconstructing the evolutionary history of deep-sea nematode taxa. Morphological evidence previously indicated that most deep-sea genera are ubiquitous and benthic community assemblages are similar worldwide [18], although a number of novel deep-sea genera had been identified [19-21]. Within our dataset, deep-sea specimens comprised a taxonomically diverse group of genera representing every major phylogenetic lineage in the Enoplida (Figure 1). Certain genera were particularly abundant in deep-sea sediments (e.g. *Halalaimus*) and isolated from most geographic locations, supporting previous conclusions from taxonomic studies.

Clade structures (Figure 1) do not reveal any concrete evidence for endemic deep-sea lineages, and deep-sea nematodes do not represent a separate, independent clade within the Nematoda. Instead, specimens are recovered as close relatives of shallow-water species. Our data suggest repeated and recent interchanges between the deep-sea and intertidal zone, supporting ideas that habitat transitions are frequent and common amongst nematodes [22]. A recent study by Van Gaever *et al. *[23] reported similar findings, observing low shallow-deep molecular divergences in *Halomonhystera *and *Terschellingia *specimens inhabiting bathyal shelf sediments. In the present study, topologies of more densely sampled genera provide evidence for multiple, independent invasions of both deep-sea and shallow water habitats. Within the genus *Halalaimus *(Clade IIIa, Figure 1), there appear to be at least 3 separate invasions of shallow habitats, and evidence for at least 3 independent colonizations of the deep-sea; two deep-sea lineages also appear to show reinvasions from shallow water. Clades I and II (Figure 1) instead support movement from shallow to deep habitats; deep-sea specimens in these clades appear to be recently derived from shallow water fauna.

This complex structure suggests multiple evolutionary origins for deep-sea nematode taxa, similar to patterns observed in isopods [10]. Although we can never unequivocally confirm an exact evolutionary history from phylogenetic topologies, the most parsimonious interpretation of our data supports both deep-sea and shallow water origins for extant Enoplid taxa. In some clades (Clades IIIa and IIIb, Figure 1), extant intertidal taxa may be originally derived from historical source populations in the deep-sea. In other taxa (Clades I and II, Figure 1) deep-sea taxa are instead nested amongst intertidal species, implying recent shallow water ancestry within these clades. Deep-sea clades additionally represent a mixture of early-branching lineages and recently derived taxa (e.g. Clade IIIa, Figure 1), supporting an evolutionary history defined by multiple invasion events. Additional data will continue to add further insight to these emerging patterns; although we aimed for intensive sampling within the Enoplida, our investigation was not an exhaustive inventory of global marine habitats and did not recover all known genera within this group.

Although we cannot label any marine lineage as 'endemic', species distributions of extant nematodes indicate that at least some taxa are not found outside the deep-sea. The genus *Acantholaimus *is rarely found in shallow water, with only one species described from intertidal sediments [24]. Morphological evidence suggests that species within this typical deep-sea group have migrated up to shallower shelf habitats in the Weddell Sea [25]; it is possible that the overall absence of *Acantholaimus *in shallow habitats could represent a secondary reduction of species in these habitats, following radiation events and speciation in the deep-sea. Although there are currently no molecular studies of *Acantholaimus *species, the present study has included representatives of another purely deep-sea genus, *Bathyeurystomina *[26]. This genus does not appear to have any recent shallow-water relatives in molecular phylogenies and appears to have split off early from other genera within the Enchelidiidae (Clade IIa, Figure 1); more extensive sampling is needed to further elucidate generic relationships within this family. Similarly, the genus *Cricohalalaimus *(Clade IIIc, Figure 1) has only been described from deep-sea sediments [19]-- molecular evidence indicates that this taxon represents a divergent lineage that is most closely related to genera *Thalassoalaimus *and *Litinium *(SSU and LSU support values >98%), even though the distinct morphology of *Cricohalalaimus *does not intuitively suggest a relationship with either group.

### Widely distributed, closely related species assemblages in the deep-sea

Regional isolation in the deep-sea has previously been implied in taxonomic studies, which typically record a high number of distinct, geographically restricted morphospecies and only rare descriptions of the same species from distant locales [1,21]. In our dataset, gene sequences from well-sampled Enoplid clades do not suggest any obvious clustering pattern according to ocean basin or collection depth (Figure 2)--phylogenetic topologies instead indicate widely distributed, closely related species assemblages (with potential conspecific status) in the deep-sea. Many genera contained specimens from distant sample sites that exhibited pairwise sequence identities >97%. Although such pairwise values are not a definitive indicator of a species, these high sequence identities suggest recent vicariance or dispersal between remote deep-sea locations. One set of Phanodermatidae specimens displayed high pairwise identities (99%) in 18 S sequences but showed signs of mitochondrial divergence; 'TCR 75' and 'Cr 72b' (Figure 2b) were collected from deep-sea sites 11,000 miles apart and exhibit sequence identities of 99% (18S), 94% (28S) and 85% (*cox1*). This pairwise identity in *cox1 *sequences exceeds interspecific divergence values reported in other nematode genera [ranging from 4.6-13.7%; 17, [27-29]], suggesting that these two nematodes represent closely related species. Five additional deep-sea Enoplids within the Phanodermatidae exhibited >99% SSU sequence identities, but our efforts failed to amplify *cox1 *from most of these specimens.

Close phylogenetic relationships were additionally observed amongst specimens from Antarctic shelf habitats (670-1406 m) and abyssal sediments in the Southern Indian Ocean (~4200 m). Within the Phanodermatidae (Figure 2b), the deep-sea sub-Antarctic specimen 'Cr 72b' exhibits a 99% (SSU) and 93% (LSU) pairwise sequence identity with Antarctic shelf specimen 'BCA 37', despite a difference of ~2800 metres in vertical depths; similar relationships were observed within the genus *Halalaimus *(Figure 2a). While LSU divergence points towards separate species, high ribosomal sequence identities nonetheless suggest a relatively recent common ancestor between these species. Although we did not sample intermediate depths outside the Southern Ocean, low genetic divergence across vertical depth may be specific to Antarctic shelf habitats; such patterns may reflect historical cycles of shelf ice formation and retreat, where periodic extinctions of shelf fauna were followed by upward migrations of deep-sea taxa [30]. (Apart from identical 18S/28 S sequences observed in *Trefusia *specimens from the Clyde estuary and the Antarctic shelf, there were no identical sequences representing both deep-sea and shallow water specimens outside the Antarctic.)

A number of benthic species are known to maintain wide geographic distributions in the deep-sea [31,32], although most of these taxa produce dispersive propagule stages or pelagic larvae that facilitate gene flow over these large distances. Marine nematodes lack any obvious propagative phases, and thus little research has been conducted on dispersal mechanisms for deep-sea species. The present study presents compelling evidence for cosmopolitan Oncholaimid species, supporting previous evidence for long-distance dispersal and broad geographic ranges in shallow-water nematodes [16,17]. Given the close evolutionary relationships between deep and shallow marine species, it seems plausible that deep-sea species retain behaviours that promote similarly broad distributions in the deep sea. Future investigations will need to sample nematodes from other continental shelf sites in order to assess whether species' large depth ranges are specific to Antarctic habitats.

### High nematode diversity in the deep-sea

Free-living marine nematodes appear to exhibit a high species diversity--a complex branching structure containing a number of divergent clades was observed within the most densely sampled nematode clades, (Clades IIIa and IIIb, Figure 1). Community assemblages in the deep-sea appear to comprise a diverse group of species; specimens representing multiple phylogenetic lineages within the same genus (presumably different species) were often present within the same geographic region, and even within the same sample site. For example, nematodes representing 3 distinct *Halalaimus *lineages were found within a single sediment core in the Southern Indian Ocean (site 15775#37, black squares in Figure 2a); at least 8 unique *Halalaimus *lineages seem to be present across all sites in the Southern Indian Ocean, despite sample areas being no more than ~500 km apart (sample codes prefaced with 'Cr' in Figure 2a). Similar patterns were observed in other well-sampled clades such as the Phanodermatidae (Figure 2b) and *Oxystomina *(Additional file 2, Figure S1).

Although this study aimed to intensively sample Enoplid taxa from each deep-sea site, our overall sampling regime only examined a limited number of sediment cores representing a very small area of the seabed (a total of 785 cm^2^). Our investigation suggests a huge diversity of species, despite this small total surface area. Furthermore, our restricted focus on the order Enoplida analysed only a specific fraction of the diverse nematode assemblages present in deep-sea sediments. Given the diversity uncovered within the Enoplida alone, the global species richness of deep-sea nematode fauna is likely to be very high.

### Cosmopolitan species in intertidal habitats

When added to other evidence of broad species ranges in nematodes [16,17], our results strongly support the existence of cosmopolitan Oncholaimid species. Although several mitochondrial haplotypes were found amongst Oncholaimid specimens possessing identical ribosomal sequences (Additional file 1, Table S1), *cox1 *variants only differed at 1-3 nucleotide positions, equating to a genetic divergence <1%. This low divergence is comparable to intraspecific variation observed in other nematode species [27]; furthermore, the same haplotype was often recovered from transatlantic locations. Potential mechanisms that may help maintain cosmopolitan distributions in Oncholaimids include water-column processes, natural rafts (vegetation masses, sea ice, marine snow) or anthropogenic transport. Hydrodynamic forces have been shown to place an important role in the transport of shallow-water meiofauna; heavy storms are known to erode sediments at depths up to 25 m and carry sediments up to 50 km away [33], while more typical tidal actions can transport meiofauna at a rate of 10 km per day as a result of erosion and passive drift [34]. Previous studies have reported an abundance of nematodes in floating mangrove detritus [35], drifting algae [36], and *Phaeocystis *'seafoam' [37], promoting the role of raft attachment as a dispersal method. Meiofauna have also been observed to aggregate in clumps of 'marine snow' [38], and transport via these small organic parcels would presumably provide nutritional sustenance during pelagic journeys. Some authors have additionally suggested that meiofauna can be transported via sea ice [33], floating pieces of rubbish [39], or even attached to birds' feet [40]. In terms of anthropogenic transport, ballast water (and its associated sediment) and fouling on vessels are known to carry meiofaunal populations across oceans during shipping operations [33]. The nematode *Pellioditis marina *is one nematode species that apparently maintains transatlantic populations, and specimens have been found floating in macroalgae rafts in the North Sea [17]; however, the frequency of long-distance dispersal events is unknown.

Behavioural evidence also suggests that Oncholaimids may be particularly adept at long-distance dispersal. *Viscosia viscosa *often inhabits the surface layers of sediment and is apparently capable of floating [41,42], increasing the probability that this species will be resuspended in the water column and passively transported. In addition, there is evidence to suggest that Oncholaimid nematodes can actively and rapidly transport themselves to suitable new habitats [41,43,44], supplementary to passive mechanisms. Our investigation was focused on a single nematode order that may be unique amongst free-living marine species; many Enoplids are large, predatory nematodes and may thus be more motile than smaller bacterial or fungal feeding nematode species. This motility may enable continued gene flow across geographically disparate sites and prevent allopatric speciation. The unique lifestyles of Enoplid nematodes may also help to explain the closely related species assemblages observed across deep-sea sites. Additional molecular studies are needed in order to assess the distribution of species (and potential for cosmopolitan ranges) in other nematode orders.

Similar patterns may additionally exist within the Trefusiidae; the observation of identical *Trefusia *species in a Scottish estuary and Antarctic shelf habitats may provide another plausible example of long-distance dispersal as well as eurybathic species distribution, although, only one specimen was recovered from the Antarctic site. Lab contamination can be ruled out, and video capture images of morphology confirm the taxonomic identity of these specimens--regardless, additional data is needed to validate this preliminary insight.

## Conclusions

This study has provided the first insight into the evolution and phylogeography of deep-sea nematode fauna, but much further work is needed. Our data suggest high species richness for marine nematodes, frequent interchanges between deep and shallow habitats, and closely related species complexes in deep-sea habitats, but data from other nematode groups (e.g. Chromadorida, Monhysterida) is needed to assess whether these patterns are broadly applicable to all nematode taxa. Despite the intensive sampling effort of this investigation, deep-sea nematodes were only isolated from a small number of sites representing a minute fraction of the seafloor. In addition, our efforts focused on relationships at the genus level, and the scope of this study was not aimed at delineating species. Future molecular investigations should be expanded to include a wider range of marine sites (shelf, bathyal, and abyssal depths), include a heavy focus on mitochondrial genes (to provide finer resolution at the species level), and represent the full taxonomic spectrum of deep-sea nematode communities.

## Methods

### Sample collection

**Figure 3:**
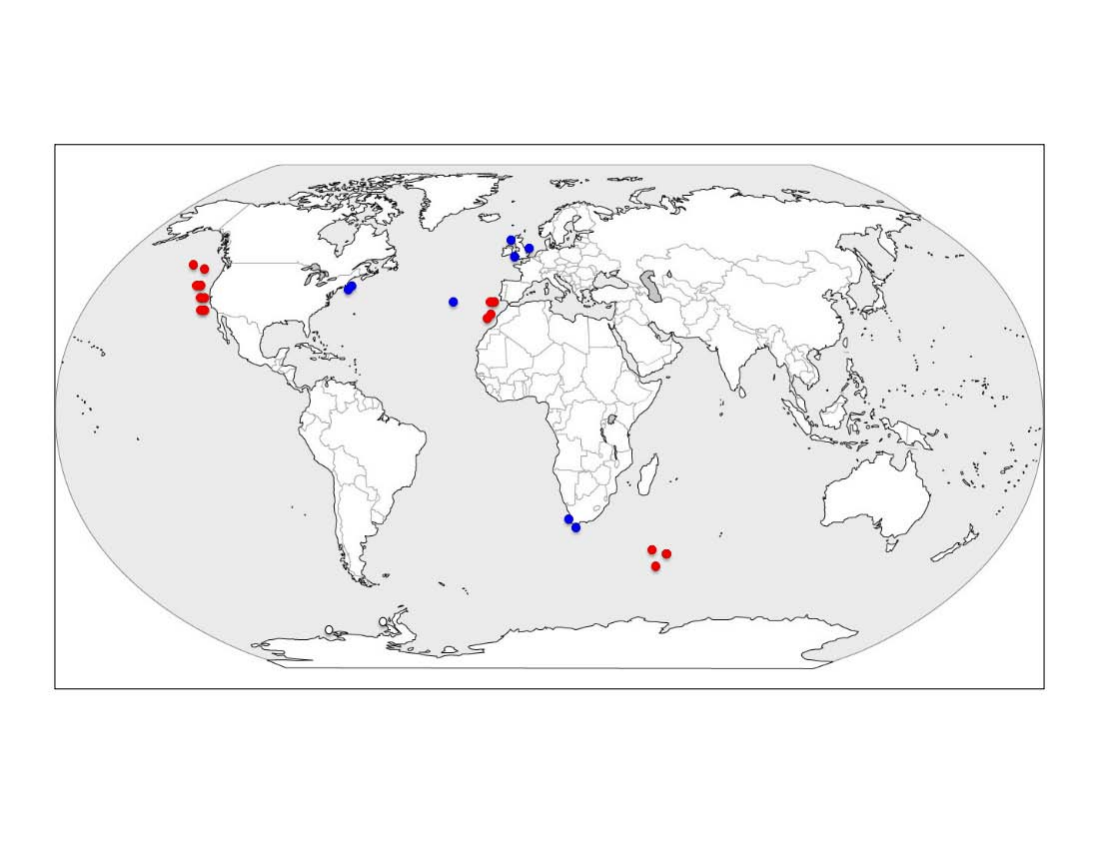
**Map outlining sampling locations utilised in this study**. Blue dots indicate intertidal sites and red dots indicate deep-sea or shelf sites.

Samples were collected from several intertidal locations as well as deep-sea sediments (Figure 3); a full list of sampling locations and collection depth is outlined in Additional file 1, Table S2. All marine sediments were immediately fixed in DESS preservative [45] using an equal ratio of preservative to sediment. The meiofauna fraction of all samples was extracted via decantation and floatation in Ludox using a 45 μm sieve according to the methods of Somerfield *et al. *[46]. Individual nematodes (representing the order Enoplida) were picked out of the meiofauna fraction using a fine wire instrument, mounted on slides, and identified to genus level; video capture images were recorded for all specimens in order to retain a voucher of morphology before specimens were destroyed for molecular analysis. Voucher images were deposited in the NemaTOL database http://nematol.unh.edu/ and are freely available in digital format.

### Isolation and sequencing of 18 S rRNA genes

Gene sequences were obtained for a total of 254 individual nematode specimens, representing 18 S rRNA (Accession numbers HM564399-HM564654), 28 S rRNA (Accession numbers HM564655-HM564910), and *cox1 *(Accession numbers HM564911-HM565012) gene sequences from a variety of Enoplid genera; Morphological IDs and corresponding accession numbers for each nematode specimen are outlined in Additional file 1, Table S3. Genomic DNA was extracted using a proteinase K digestion [47]; individual specimens were picked into microcentrifuge tubes containing 25 μl distilled water, followed by the addition of 25 μl lysis buffer (containing 0.2 M NaCl, 0.2 M Tris-HCl (pH 8.0), 1% β-mercaptoethanol and 800 μg/ml proteinase K). Tubes were incubated for 2 h at 65°C and 750 rpm in an Eppendorf Thermomixer (Eppendorf, Hamburg, Germany), followed by a final 5 min at 100°C and 750 rpm. Final lysates were stored at -20°C. All PCR reactions were conducted using a DyNAzyme EXT PCR kit (New England Biolabs, Ipswich, MA, USA), with a final reaction volume of 25.75 μl. Each reaction contained 2 μl of nematode genomic DNA, 18.25 μl sterile water, 0.4 mM of each primer (Integrated DNA technologies, Coralville, IA, USA) 2.5 μl 10X DyNAzyme EXT Buffer containing MgCl_2 _(final reaction volume 1.5 mM MgCl2), 0.5 μl dNTP mix containing 10 μM each nucleotide, and 0.5 μl DyNAzyme EXT DNA polymerase (0.5 enzyme units in final reaction volume). Nearly full-length 18 S rRNA gene sequences (~1600 bp) were amplified from all nematodes using primer sets SSU_F_04 and SSU_R_26, SSU_F_22 and SSU_R_13, and SSU_F_24_1 and SSU_R_81 [48,49] (primer sequences available at http://www.nematodes.org/research/barcoding/sourhope/nemoprimers.html). The D2/D3 expansion segment of the 28 S rRNA gene (~600 bp) was additionally amplified from all specimens using primers D2Ab and D3B [50]. Primers JB3 and JB5 [27] were used to amplify a fragment of the mitochondrial *cox1 *gene (393 bp) from a subset of 99 specimens. The following PCR profile was used to amplify all primer sets: 94°C for 5 min followed by 35 cycles of denaturation at 94°C for 30 seconds, annealing at 54°C for 45 seconds, extension at 72°C for 2 minutes, with a final extension of 72°C for 10 min. All PCR products were visualized on a 1.5% agarose gel containing Ethidium Bromide.

Successful PCR reactions were purified using a QIAquick PCR purification kit (QIAGEN, Valencia, CA, USA). Sequencing reactions were carried out using a BigDye Terminator v3.1 cycle sequencing kit (Applied Biosystems, Foster City, CA, USA), with individual sequencing reactions having a final volume of 10 μl; all PCR products were sequenced in both directions. Each reaction contained 3 μl 5X ABI sequencing buffer, 2 μl of 2 μM forward or reverse primer, 1 μl BigDye Terminator v1.1, and either 2 μl or 4 μl of purified PCR product. Sequencing reactions were carried out using the following thermal profile: 96°C for 1 minute followed by 25 cycles of 96°C for 10 seconds, 50°C for 5 seconds, and 60°C for 4 minutes. Cycle-sequence products were purified via ethanol precipitation and sequenced using an ABI 3130 genetic analyzer.

### Sequence Alignment and Phylogenetic Analysis

Pre-aligned structural alignments of 18 S and 28 S rRNA genes were downloaded from release 98 of the SILVA rRNA database [51] and imported into the ARB software suite [52]. Enoplid sequences generated during this investigation were incorporated into nematode secondary structure alignments via the Positional Tree (PT) Server function in the ARB software suite. Alignment quality was assessed by first constructing Neighbour-Joining trees in ARB; some manual editing was necessary to ensure that all secondary structure motifs were properly aligned. Short sequences (< 1000 bp) and sequences of dubious quality were removed from the alignment. A subset of Dorylaimid nematodes were chosen and aligned as outgroup taxa in ribosomal phylogenies, representing close relatives of the subclass Enoplia [53]. Final 18 S gene alignments contained contained 354 unique sequences representing 37 genera from the order Enoplida; final LSU datasets contained 280 Enoplid sequences. Sequence from the Rhabditid nematode *Pellioditis marina *were utilised as outgroup taxa in *cox1 *datasets; final mitochondrial alignments contained 105 taxa and were aligned using ClustalW in MEGA 4.0 [54].

Gene alignments were used to construct Maximum Likelihood trees using Randomized Axelerated Maximum Likelihood (RAxML) version 7.04 [55,56], hosted at the Vital-IT unit of the Swiss Institute of Bioinformatics http://phylobench.vital-it.ch/raxml-bb/. Support values were generated from RAxML runs using 100 bootstrap replicates. Bayesian inference was used to supplement topological inferences. Datasets were run for up to 4,000,000 generations using the GTR+I+G model of nucleotide substitution, 4 MCMC chains, and a heating temperature of 0.06; in all runs, the first 25% of sampled trees were discarded as burn-in. Data was submitted to the CIPRES project cluster hosted at the University of California, San Diego and analysed using MrBayes3.2 http://www.phylo.org/sub_sections/portal/. In both methods, ribosomal data was analysed using partitions according to secondary structure (Stems/Loops), while mitochondrial data was run using codon partitions.

### Analysis of sequence divergence

Evolutionary relationships were further investigated by computing pairwise sequence comparisons and analysis of isolation by distance within certain genera. Distance matrices were computed in MEGA v4.0 [54] using p-distances, and pairwise sequence identities were subsequently compared to observed phylogenetic topologies.

## Authors' contributions

HMB carried out nematode extraction and sequencing, sequence alignment, tree building, and drafted the manuscript. DHL participated in sequence alignment and tree building. WKT participated in nematode sequencing and aided in designing the study. PJDL conceived the study and participated in its design and coordination. All authors read and approved the final manuscript.

## Supplementary Material

Additional file 1 **Supplementary tables listing groups of nematodes with identical gene sequences, sample sites utilized in this study, and accession numbers of all gene sequences obtained**. **Table S1 - **Groups of Enoplid nematodes exhibiting identical ribosomal sequences. All specimens within each box possess identical copies of both SSU and LSU gene sequences; maximum distance between specimens' collection sites is listed per group. Mitochondrial sequences were additionally isolated from taxa in bold, with letters in brackets representing different *cox1 *haploypes. **Table S2 - **Geographic data and collection depth of all sample sites used in this study. Short location codes were used to identify nematodes from different geographic locations after individual specimens were digested for molecular work. **Table S3 - **Genbank Accession numbers of all 18 S, 28 S and *cox1 *sequences amplified during this investigation Click here for file

Additional file 2 **Expanded Bayesian 18 S Phylogeny of the Enoplida**. **Figure S1 **- Bayesian phylogeny based on SSU data displaying the habitats of marine nematodes within the Enoplida, expanded to show all taxa. Black taxa = shallow-water, red taxa = deep-sea Southern Indian Ocean, blue taxa = deep-sea Pacific, and yellow taxa = Antarctic shelf. Collection depths listed after all deep-sea specimens, and sample codes correspond to sample sites outlined in Table 1. Click here for file
