# Supplementary material for: Low endemism, continued deep-shallow interchanges, and evidence for cosmopolitan distributions in free-living marine nematodes (order Enoplida)
Source: BMC Evol Biol. 2010 Dec 18;10:389. doi: 10.1186/1471-2148-10-389 (PMC3022606; doi:10.1186/1471-2148-10-389)
Supplement: Additional file 1 — Supplementary tables listing groups of nematodes with identical gene sequences, sample sites utilized in this study, and accession numbers of all gene sequences obtained. Table S1 - Groups of Enoplid nematodes exhibiting identical ribosomal sequences. All specimens within each box possess identical copies of both SSU and LSU gene sequences; maximum distance between specimens' collection sites is listed per group. Mitochondrial sequences were additionally isolated from taxa in bold, with letters in brackets representing different cox1 haploypes. Table S2 - Geographic data and collection depth of all sample sites used in this study. Short location codes were used to identify nematodes from different geographic locations after individual specimens were digested for molecular work. Table S3 - Genbank Accession numbers of all 18 S, 28 S and cox1 sequences amplified during this investigation [file 1471-2148-10-389-S1.PDF]

Table S1

Sequence ID	Sample Site	Distance
AUK 10 *Viscosia*	Appledore, Torridge Estuary ,UK	13 to 4,781 km between sites
HCL 5 Oncholaimidae	Helensburgh, Clyde Estuary, UK	
HCL 7 Oncholaimidae	Helensburgh, Clyde Estuary, UK	
HCL 9 *Viscosia*	Helensburgh, Clyde Estuary, UK	
HCL 2 Oncholaimidae	Helensburgh, Clyde Estuary, UK	
HCL 10 *Viscosia*	Helensburgh, Clyde Estuary, UK	
HCL 11 *Viscosia*	Helensburgh, Clyde Estuary, UK	
HCL 12 Oncholaimidae	Helensburgh, Clyde Estuary, UK	
HCL 15 *Viscosia*	Helensburgh, Clyde Estuary, UK	
HCL 24 *Viscosia*	Helensburgh, Clyde Estuary, UK	
HCL 27 *Viscosia*	Helensburgh, Clyde Estuary, UK	
**HUK 1 Oncholaimidae [A]**	**Helensburgh, Clyde Estuary, UK**	
LUK 3 *Viscosia*	Lunderston, Clyde Estuary, UK	
**OUS 1 Oncholaimidae [A]**	**Odiorne Point, New Hampshire, USA**	
OUS 14 Oncholaimidae	Odiorne Point, New Hampshire, USA	
OUS 21 Oncholaimidae	Odiorne Point, New Hampshire, USA	
OUS 9 Oncholaimidae	Odiorne Point, New Hampshire, USA	
**BUS 2 *Oncholaimus* [C]**	**Barnstaple, Massachusetts, USA**	32 to 12,485 km between sites
**BUS 3 *Oncholaimus* [B]**	**Barnstaple, Massachusetts, USA**	
BUS 5 *Oncholaimus*	Barnstaple, Massachusetts, USA	
**BUS 7 *Oncholaimus* [B]**	**Barnstaple, Massachusetts, USA**	
**NUS 4 *Oncholaimus* [C]**	**Nauset, Massachusetts, USA**	
**NUS 6 *Oncholaimus* [C]**	**Nauset, Massachusetts, USA**	
NUS 7 *Oncholaimus*	Nauset, Massachusetts, USA	
**DBA 4 *Oncholaimus* [B]**	**Dolphin Beach, Cape Agulhus, South Africa**	
**SBA 2 *Oncholaimus* [C]**	**Struis Bay, South Africa**	
**SBA 3 *Oncholaimus* [B]**	**Struis Bay, South Africa**	
**SBA 5 *Oncholaimus* [B]**	**Struis Bay, South Africa**	
NAR 2 *Enoplolaimus*	Narragansett, Rhode Island, USA	5 to 191 km between sites
**NAR 8 *Enoplolaimus* [D]**	**Narragansett, Rhode Island, USA**	
**SUS 1 *Enoplolaimus* [D]**	**Scarborough, Rhode Island, USA**	
SUS 10 *Enoplolaimus*	Scarborough, Rhode Island, USA	
SUS 15 *Enoplolaimus*	Scarborough, Rhode Island, USA	
**SUS 6 *Enoplolaimus* [D]**	**Scarborough, Rhode Island, USA**	
WUS 5 *Enoplolaimus*	Wallis Sands, New Hampshire, USA	
**NAR 1 *Enoplolaimus* [E]**	**Narragansett, Rhode Island, USA**	5 km between sites
NAR 5 *Enoplolaimus*	Narragansett, Rhode Island, USA	
NAR 9 *Enoplolaimus*	Narragansett, Rhode Island, USA	
**SUS 2 *Enoplolaimus* [E]**	**Scarborough, Rhode Island, USA**	
**SUS 21 *Enoplolaimus* [E]**	**Scarborough, Rhode Island, USA**	
BUS 15 *Tripyloides*	Barnstaple, Massachusetts, USA	32 km between sites
NUS 14 *Tripyloides*	Nauset, Massachusetts, USA	
NUS 41 *Tripyloides*	Nauset, Massachusetts, USA	

NUS 40 *Anoplostoma*<br>OUS 3 *Anoplostoma*<br>OUS 5 *Anoplostoma*<br>OUS 6 *Anoplostoma*<br>OUS 7 *Anoplostoma*<br>OUS 8 *Anoplostoma*	Nauset, Massachusetts, USA<br>Odiorne Point, New Hampshire, USA<br>Odiorne Point, New Hampshire, USA<br>Odiorne Point, New Hampshire, USA<br>Odiorne Point, New Hampshire, USA<br>Odiorne Point, New Hampshire, USA	152 km<br>between sites
BCA 10 *Trefusia*<br>LCL 1 *Trefusia*<br>LCL 2 *Trefusia*<br>LCL 3 *Trefusia*<br>LCL 4 *Trefusia*<br>LCL 7 *Trefusia*<br>LCL 8 *Trefusia*	670m deep-sea Antarctic, sample site BC 470<br>Lunderston, Clyde Estuary, UK<br>Lunderston, Clyde Estuary, UK<br>Lunderston, Clyde Estuary, UK<br>Lunderston, Clyde Estuary, UK<br>Lunderston, Clyde Estuary, UK<br>Lunderston, Clyde Estuary, UK	14,996 km<br>between sites
Cr 55 *Halalaimus*<br>Cr 83b *Halalaimus*	4202m deep-sea sub-Antarctic, sample site 15775#3<br>4192m deep-sea sub-Antarctic, sample site 15775#33	13 km<br>between sites
BCA 1 *Syringolaimus*<br>BCA 2 *Syringolaimus*<br>BCA 31 *Syringolaimus*<br>BCA 47 *Syringolaimus*<br>BCA 5 *Syringolaimus*<br>BCA 6 *Syringolaimus*	670m deep-sea Antarctic, sample site BC 470<br>670m deep-sea Antarctic, sample site BC 470<br>1406m deep-sea Antarctic, sample site BC 477<br>1406m deep-sea Antarctic, sample site BC 477<br>670m deep-sea Antarctic, sample site BC 470<br>670m deep-sea Antarctic, sample site BC 470	1,105 km<br>between sites
BCA 23 *Oxystomina*<br>BCA 42 *Oxystomina*	1120m deep-sea Antarctic, sample site BC 476<br>1406m deep-sea Antarctic, sample site BC 477	43 km<br>between sites
Cr 73a *Chaetonema*<br>Cr 76a *Chaetonema*<br>Cr 83a *Chaetonema*<br>Cr 84b *Chaetonema*	4197m deep-sea sub-Antarctic, sample site 15775#32<br>4202m deep-sea sub-Antarctic, sample site 15775#25<br>4202m deep-sea sub-Antarctic, sample site 15775#25<br>4192m deep-sea sub-Antarctic, sample site 15775#33	3 to 24 km<br>between sites
TCR 173 Phanodermatidae<br>TCR 188 *Phanodermopsis*	2720m, deep-sea Pacific, sample site 817 nem<br>2694m deep-sea Pacific, sample site 856 nem	1 km<br>between sites
TCR 143 *Thoracostomopsidae*<br>TCR 158 Thoracostomopsidae	3855m deep-sea Pacific, sample site 712 nem<br>2720m deep-sea Pacific, sample site 817 nem	26 km<br>between sites

Table S2

Location	Coded As	Latitude	Longitude	Depth	Collected
Appledore, Torridge Estuary, UK	AUK/BAUK	51° 1' 54” N	4° 12' 12” W	Intertidal	19-Feb-08
Llansteffan, Towy Estuary, UK	LUK	51° 47' 18" N	4° 22' 15" W	Intertidal	20-Feb-08
All Hallows, Thames Estuary, UK	HUK	51° 28’ 52.56"N	0° 38’ 47.58" E	Intertidal	21-Jun-08
Shoebury Ness, Thames Estuary, UK	SBN	51° 31’ 40.32" N	0° 48' 43.44" E	Intertidal	18-Jun-08
Helensburgh, Clyde Estuary, UK	HCL	56° 0' 10.97" N	4° 44' 12.87" W	Intertidal	30-Aug-08
Lunderston, Clyde Estuary, UK	LCL	55° 55' 15.27" N	4° 52' 38.51" W	Intertidal	30-Aug-08
Barnstaple, Massachusetts, USA	BUS	41° 50' 35.48" N	69° 57' 4.62" W	Intertidal	28-Mar-08
Nauset, Massachusetts, USA	NUS	41° 42' 19.70" N	70° 18' 5.87" W	Intertidal	28-Mar-08
Narragansett, Rhode Island, USA	NAR	41° 26' 5.96" N	71° 27' 19.43" W	Intertidal	27-Jun-08
Scarborough, Rhode Island, USA	SUS	41° 23' 26.35" N	71° 28' 16.52" W	Intertidal	27-Jun-08
Odiorne Point, New Hampshire, USA	OUS	43° 2' 54.62" N	70° 43' 47.0" W	Intertidal	19-Jun-08
Wallis Sands State Beach, New Hampshire, USA	WUS	43° 1' 37.44" N	70° 43' 41.82" W	Intertidal	19-Jun-08
Porto Pim, Faial island, Azores	PPA	38° 31' 25” N	28° 37' 32” W	Intertidal	13-Sep-08
Dolphin Beach, Cape Agulhus, South Africa	DBA	33° 48' 44.02" S	18° 28' 10.73" E	Intertidal	26-Jan-07
Struis Bay, South Africa	SBA	34° 47' 24.82" S	20° 2' 51.29" E	Intertidal	23-Jan-07
Erosional Fairway, Seine Abyssal Plain, Atlantic Ocean, JC27-22#1	JCC	35° 33' 16.8" N	9° 41' 55.2" W	4321 m	15-Aug-08
Inside Scour, Seine Abyssal Plain, Atlantic Ocean JC27-25#2	JCC	35° 44' 45" N	9° 59' 16.2" W	4630 m	16-Aug-08
Sao Vicente Canyon Mouth, Atlantic Ocean, JC27-29	JCC	36° 13' 3.6" N	10° 1' 49.2" W	4878 m	17-Aug-08
Cascais canyon mouth, Atlantic Ocean, JC27-43	JCC	38° 21' 39.6" N	9° 59' 4.8" W	4572 m	22-Aug-08
Cascais canyon, Atlantic Ocean, JC27-45	JCC	38° 23' 18" N	10° 24' 7.8" W	4835 m	23-Aug-08
Off coast California, Core 112 Nem	TCR	43° 59' 49.98" N	130° 23' 36" W	3260 m	16-Sep-08
Off coast California, Core 221 Nem	TCR	42° 33' 28.32" N	132° 0' 40.2" W	3605 m	18-Sep-08
Off coast California, Core 312 Nem	TCR	39° 59' 58.2" N	125° 52' 27.24" W	3673 m	20-Sep-08
Off coast California, Core 418 Nem	TCR	39° 59 '52.86" N	125° 26' 36.06" W	2730 m	21-Sep-08
Off coast California, Core 518 Nem	TCR	36° 47' 17.28" N	123° 41' 28.86" W	3673 m	23-Sep-08
Off coast California, Core 617 Nem	TCR	36° 40 '52.2" N	122° 49' 36.6" W	2692 m	24-Sep-08
Off coast California, Core 712 Nem	TCR	32° 52' 39.42" N	120° 36' 30.84" W	3855 m	27-Sep-08
Off coast California, Core 817 Nem	TCR	32° 47' 49.14" N	120° 22' 16.02" W	2720 m	28-Sep-08
Off coast California, Core 856 Nem	TCR	32° 47' 54.24" N	120° 22' 20.7" W	2694 m	30-Sep-08
Off coast California, Core 861 Nem	TCR	32° 47' 52.32" N	120° 22' 18.36" W	2695 m	1-Oct-08
Bellinghausen Sea, off Antarctica, Biopearl II BC 470	BCA	69° 05' 18" S	76° 23' 21" W	670 m	29-Feb-08
Pine Island Bay, inner shelf basin, off Antarctica, Biopearl II BC 476	BCA	74° 29' 00" S	104° 25' 00" W	1120 m	6-Mar-08
Pine Island Bay, inner shelf basin, off Antarctica, Biopearl II BC 477	BCA	74° 21' 47" S	104° 40' 19" W	1406 m	6-Mar-08
Southern Indian Ocean, off Crozet islands, CROZET core 15772#2	Cr	44° 29' 40" S	50° 0' 54" E	2908 m	8-Dec-05
Southern Indian Ocean, off Crozet islands, CROZET core 15773#18	Cr	45° 52' 57" S	56° 23' 46" E	4186 m	15-Dec-05
Southern Indian Ocean, off Crozet islands, CROZET core 15773#21	Cr	45° 53' 40" S	56° 24' 23" E	4193 m	15-Dec-05
Southern Indian Ocean, off Crozet islands, CROZET core 15773#27	Cr	45° 53' 33" S	56° 25' 1" E	4210 m	18-Dec-05
Southern Indian Ocean, off Crozet islands, CROZET core 15773#31	Cr	45° 53' 48" S	56° 25' 46" E	4200 m	20-Dec-05
Southern Indian Ocean, off Crozet islands, CROZET core 15775#3	Cr	49° 3' 38" S	51° 14' 12" E	4202 m	27-Dec-05
Southern Indian Ocean, off Crozet islands, CROZET core 15775#25	Cr	49° 4' 31" S	51° 13' 7" E	4202 m	3-Jan-06
Southern Indian Ocean, off Crozet islands, CROZET core 15775#32	Cr	49° 2' 30" S	51° 12' 50" E	4197 m	4-Jan-06
Southern Indian Ocean, off Crozet islands, CROZET core 15775#33	Cr	49° 1' 58" S	51° 13' 58" E	4192 m	4-Jan-06
Southern Indian Ocean, off Crozet islands, CROZET core 15775#37	Cr	49° 1' 52" S	51° 14' 5" E	4192 m	5-Jan-06

Table S3

Seq. ID	Taxonomic ID	SSU	LSU	Cox1
AUK 1	*Tripyloides sp.*			HM564911
AUK 7	*Tripyloides sp.*			HM564915
AUK 10	*Viscosia sp.*	HM564399	HM564655	
AUK 13	*Calyptronema sp.*	HM564400	HM564656	HM564912
AUK 14	*Oxystomina sp.*	HM564401	HM564657	
AUK 18	*Calyptronema sp.*			HM564913
AUK 23	*Oncholaimus sp.*	HM564402	HM564658	HM564914
AUK 35	*Oncholaimus sp.*	HM564474	HM564730	
AUK 36	*Oncholaimus sp.*	HM564475	HM564731	
AUK 45	*Tripyloides sp.*	HM564476	HM564732	
BAUK 9	*Oxystomina sp.*	HM564403	HM564659	
BCA 1	*Syringolaimus sp.*	HM564477	HM564733	
BCA 2	*Syringolaimus sp.*	HM564485	HM564741	
BCA 3	*Pareurystomina*	HM564491	HM564746	
BCA 5	*Syringolaimus sp.*	HM564500	HM564755	
BCA 6	*Syringolaimus sp.*	HM564501	HM564756	
BCA 10	*Trefusia sp.*	HM564478	HM564734	
BCA 12	*Halalaimus sp.*	HM564479	HM564735	
BCA 14	*Mesacanthion/Paramesacanthion sp.*	HM564480	HM564736	
BCA 15	*Oxystomina sp.*	HM564481	HM564737	
BCA 16	*Halalaimus sp.*	HM564482	HM564738	
BCA 17	*Halalaimus sp.*	HM564483	HM564739	
BCA 19	*Mesacanthion/Paramesacanthion sp.*	HM564484	HM564740	
BCA 20	Phanodermatidae sp.	HM564486	HM564742	
BCA 21	*Oxystomina sp.*	HM564487	HM564881	
BCA 22	*Oxystomina sp.*	HM564488	HM564743	
BCA 23	*Oxystomina sp.*	HM564489	HM564744	
BCA 25	*Halalaimus sp.*	HM564490	HM564745	HM564934
BCA 26	*Oncholaimus sp.*			HM564935
BCA 31	*Syringolaimus sp.*	HM564492	HM564747	
BCA 32	Phanodermatidae sp.	HM564493	HM564748	
BCA 35	*Oxystomina sp.*	HM564494	HM564749	
BCA 37	Phanodermatidae sp.	HM564495	HM564750	HM564936
BCA 38	*Halalaimus sp.*	HM564496	HM564751	
BCA 40	*Bathyeurystomina sp.*			HM564937
BCA 41	*Syringolaimus sp.*	HM564497	HM564752	
BCA 42	*Oxystomina sp.*	HM564498	HM564753	HM564938
BCA 47	*Syringolaimus sp.*	HM564499	HM564754	
BUS 1	*Oncholaimus sp.*	HM564404	HM564660	HM564916

BUS 2	*Oncholaimus sp.*	HM564406	HM564662	HM564917
BUS 3	*Oncholaimus sp.*	HM564408	HM564664	HM564918
BUS 4	*Oncholaimus sp.*	HM564409	HM564665	HM564919
BUS 5	*Oncholaimus sp.*	HM564410	HM564666	HM564920
BUS 7	*Oncholaimus sp.*	HM564411	HM564667	HM564921
BUS 15	*Tripyloides sp.*	HM564405	HM564661	
BUS 21	*Anoplostoma sp.*	HM564407	HM564663	
Cr 1	Thoracostomopsidae *sp.*	HM564412	HM564669	HM564922
Cr 3	*Phanodermopsis sp.*	HM564413	HM564668	HM564923
Cr 4	*Halalaimus sp.*			HM564924
Cr 7	*Halalaimus sp.*	HM564414	HM564687	
Cr 9	*Halalaimus sp.*	HM564415	HM564688	
Cr 11	*Halalaimus sp.*	HM564502	HM564810	
Cr 13	*Halalaimus sp.*	HM564503	HM564811	
Cr 18 b	*Mesacanthion/ Paramesacanthion sp.*	HM564504	HM564812	
Cr 19 b	*Phanodermopsis sp.*	HM564505	HM564813	
Cr 20 b	*Halalaimus sp.*	HM564506	HM564814	
Cr 21 b	Comesomatidae	HM564507	HM564815	
Cr 24 b	*Metaparoncholaimus/Meyersia sp.*	HM564508	HM564908	HM564939
Cr 26	Phanodermatidae sp.	HM564509	HM564816	
Cr 33	*Phanodermopsis sp.*	HM564510	HM564817	
Cr 34	*Mesacanthion/ Paramesacanthion sp.*	HM564511	HM564818	
Cr 35	*Halalaimus sp.*	HM564512	HM564819	
Cr 38	Anticomidae sp.	HM564513	HM564820	
Cr 54	*Phanodermopsis sp.*	HM564514	HM564821	
Cr 55	*Halalaimus sp.*	HM564515	HM564822	
Cr 56	*Phanodermopsis sp.*	HM564516	HM564823	
Cr 59	*Halalaimus sp.*	HM564517	HM564824	HM564940
Cr 60	*Halalaimus sp.*	HM564518	HM564825	
Cr 61	*Halalaimus sp.*	HM564519	HM564826	
Cr 62	*Halalaimus sp.*	HM564520	HM564827	
Cr 63	*Halalaimus sp.*	HM564521	HM564882	
Cr 64	*Halalaimus sp.*	HM564522	HM564883	
Cr 66	*Phanodermopsis sp.*	HM564523	HM564884	HM564941
Cr 68	*Phanodermopsis sp.*	HM564524	HM564885	HM564942
Cr 72a	*Halalaimus sp.*	HM564526	HM564887	
Cr 73a	*Chaetonema sp.*	HM564528	HM564828	
Cr 74a	*Halalaimus sp.*	HM564530	HM564890	
Cr 76a	*Chaetonema sp.*	HM564533	HM564893	
Cr 77a	*Oxystomina sp.*	HM564535	HM564895	
Cr 78a	*Bathyeurystomina sp.*	HM564537	HM564897	
Cr 80a	*Oxystomina sp.*	HM564538	HM564898	

Cr 82a	*Halalaimus sp.*	HM564540	HM564900	
Cr 83a	*Chaetonema sp.*	HM564542	HM564901	
Cr 85a	*Halalaimus sp.*	HM564545	HM564904	
Cr 71b	*Phanodermopsis sp.*	HM564525	HM564886	
Cr 72b	Phanodermatidae sp.	HM564527	HM564888	HM564943
Cr 73b	*Halalaimus sp.*	HM564529	HM564889	
Cr 74b	*Halalaimus sp.*	HM564531	HM564891	
Cr 75b	*Halalaimus sp.*	HM564532	HM564892	
Cr 76b	*Oxystomina sp.*	HM564534	HM564894	
Cr 77b	*Halalaimus sp.*	HM564536	HM564896	
Cr 80b	*Bathyeurystomina sp.*	HM564539	HM564899	
Cr 82b	Thoracostomopsidae sp.	HM564541	HM564909	HM564944
Cr 83b	*Halalaimus sp.*	HM564543	HM564902	
Cr 84b	*Chaetonema sp.*	HM564544	HM564903	
Cr 85b	*Halalaimus sp.*	HM564546	HM564905	
Cr 86	*Halalaimus sp.*	HM564547	HM564906	
Cr 87	*Oxystomina sp.*	HM564548	HM564907	
DBA 1	*Enoploides sp.*	HM564549	HM564757	HM564945
DBA 2	*Enoploides sp.*	HM564550	HM564758	HM564946
DBA 3	*Enoploides sp.*	HM564552	HM564760	
DBA 4	*Oncholaimus sp.*	HM564553	HM564761	HM564947
DBA 5	*Enoploides sp.*	HM564554	HM564762	HM564948
DBA 6	*Enoploides sp.*	HM564555	HM564764	HM564949
DBA 7	*Enoploides sp.*	HM564556	HM564763	HM564950
DBA 21	*Enoplus sp.*	HM564551	HM564759	
HCL 2	Oncholaimidae sp.	HM564561	HM564769	
HCL 5	Oncholaimidae sp.	HM564568	HM564776	
HCL 7	Oncholaimidae sp.	HM564569	HM564777	
HCL 8	Oncholaimidae sp.			HM564952
HCL 9	*Viscosia sp.*	HM564570	HM564778	
HCL 10	*Viscosia sp.*	HM564557	HM564765	
HCL 11	*Viscosia sp.*	HM564558	HM564766	
HCL 12	Oncholaimidae sp.	HM564559	HM564767	
HCL 15	*Viscosia sp.*	HM564560	HM564768	
HCL 20	*Oxystomina sp.*	HM564562	HM564770	
HCL 21	*Oxystomina sp.*	HM564563	HM564771	
HCL 23	Oncholaimidae sp.	HM564564	HM564772	HM564951
HCL 24	*Viscosia sp.*	HM564565	HM564773	
HCL 27	*Viscosia sp.*	HM564566	HM564774	
HCL 32	*Oxystomina sp.*	HM564567	HM564775	
HUK 1	Oncholaimidae sp.	HM564416	HM564689	HM564985
JCC 4	*Anticoma sp.*			HM564954

JCC 23	Phanodermatidae sp.	HM564571	HM564779	
JCC 29	Anticomidae *sp.*	HM564572	HM564829	
JCC 37	*Enoplolaimus sp.*			HM564953
JCC 52	Phanodermatidae sp.	HM564573	HM564780	HM564955
JCC 59	*Phanodermopsis sp.*	HM564574	HM564781	HM564956
JCC 79	*Anticoma sp.*			HM564957
JCC 89	*Phanodermopsis sp.*	HM564575	HM564782	
LCL 1	*Trefusia sp.*	HM564576	HM564783	
LCL 2	*Trefusia sp.*	HM564578	HM564785	
LCL 3	*Trefusia sp.*	HM564581	HM564788	HM564960
LCL 4	*Trefusia sp.*	HM564582	HM564789	
LCL 5	*Bathylaimus sp.*	HM564583	HM564790	HM564961
LCL 7	*Trefusia sp.*	HM564584	HM564791	HM564962
LCL 8	*Trefusia sp.*	HM564585	HM564792	
LCL 9	*Bathylaimus sp.*	HM564586	HM564793	HM564963
LCL 19	*Bathylaimus sp.*	HM564577	HM564784	HM564958
LCL 20	Oncholaimidae sp. (Viscosia sp.)	HM564579	HM564786	
LCL 21	*Bathylaimus sp.*	HM564580	HM564787	HM564959
LUK 1	*Viscosia sp.*	HM564417	HM564670	
LUK 3	*Viscosia sp.*	HM564419	HM564672	
LUK 6	*Halalaimus sp.*	HM564420	HM564673	
LUK 7	*Calyptronema sp.*	HM564421	HM564674	HM564926
LUK 12	*Calyptronema sp.*	HM564418	HM564671	HM564925
NAR 1	*Enoplolaimus sp.*	HM564422	HM564690	HM564986
NAR 2	*Enoplolaimus sp.*	HM564427	HM564695	HM564990
NAR 4	*Oncholaimus sp.*	HM564429	HM564697	
NAR 5	*Enoplolaimus sp.*	HM564430	HM564698	HM564991
NAR 6	*Chaetonema sp.*	HM564431	HM564699	HM564992
NAR 7	*Oncholaimus sp.*	HM564432	HM564700	HM564993
NAR 8	*Enoplolaimus sp.*	HM564433	HM564701	HM564994
NAR 9	*Enoplolaimus sp.*	HM564434	HM564702	HM564995
NAR 11	*Bathylaimus sp.*	HM564423	HM564691	HM564987
NAR 14	*Bathylaimus sp.*	HM564424	HM564692	HM564988
NAR 15	*Bathylaimus sp.*	HM564425	HM564693	
NAR 16	*Oncholaimus sp.*	HM564426	HM564694	HM564989
NAR 20	*Bathylaimus sp.*	HM564428	HM564696	
NUS 1	*Pareurystomina sp.*	HM564435	HM564675	
NUS 2	*Oncholaimus sp.*	HM564438	HM564678	HM564928
NUS 3	*Oxystomina sp.*	HM564440	HM564680	
NUS 4	*Oncholaimus sp.*	HM564441	HM564681	HM564930
NUS 5	*Oncholaimus sp.*	HM564444	HM564684	HM564931

NUS 6	*Oncholaimus sp.*	HM564445	HM564685	HM564932
NUS 7	*Oncholaimus sp.*	HM564446	HM564686	HM564933
NUS 10	*Oncholaimus sp.*			HM564927
NUS 11	*Bathylaimus sp.*	HM564436	HM564676	
NUS 14	*Tripyloides sp.*	HM564437	HM564677	
NUS 21	*Oxystomina sp.*	HM564439	HM564679	HM564929
NUS 40	*Anoplostoma*	HM564442	HM564682	
NUS 41	*Tripyloides sp.*	HM564443	HM564683	
OUS 1	Oncholaimidae sp.	HM564447	HM564703	HM564996
OUS 2	*Oncholaimus sp.*	HM564450	HM564706	HM564998
OUS 3	*Anoplostoma sp.*	HM564453	HM564709	HM564999
OUS 4	*Halalaimus sp.*	HM564454	HM564710	
OUS 5	*Anoplostoma sp.*	HM564455	HM564711	HM565000
OUS 6	*Anoplostoma sp.*	HM564456	HM564712	HM565001
OUS 7	*Anoplostoma sp.*	HM564457	HM564713	
OUS 8	*Anoplostoma sp.*	HM564458	HM564714	
OUS 9	Oncholaimidae sp.	HM564459	HM564715	
OUS 10	*Enoploides sp.*	HM564448	HM564704	
OUS 14	Oncholaimidae sp.	HM564449	HM564705	HM564997
OUS 21	Oncholaimidae sp.	HM564451	HM564707	
OUS 22	*Halalaimus sp.*	HM564452	HM564708	
PPA 1	*Enoplolaimus sp.*			HM564964
PPA 3	*Enoplolaimus sp.*			HM564965
PPA 5	*Enoplolaimus sp.*			HM564966
PPA 7	*Enoplus sp.*	HM564587	HM564794	HM564967
SBA 1	*Halalaimus sp.*	HM564588	HM564795	
SBA 2	*Oncholaimus sp.*	HM564592	HM564799	HM564970
SBA 3	*Oncholaimus sp.*	HM564593	HM564800	HM564971
SBA 5	*Oncholaimus sp.*	HM564594	HM564801	HM564972
SBA 7	Thoracostomopsidae sp.			HM564973
SBA 8	Thoracostomopsidae sp.			HM564974
SBA 9	Thoracostomopsidae sp.			HM564975
SBA 10	*Halalaimus sp.*	HM564589	HM564796	
SBA 12	*Halalaimus sp.*	HM564590	HM564797	
SBA 13	Thoracostomopsidae sp.	HM564591	HM564798	HM564968
SBA 14	Thoracostomopsidae sp.			HM564969
SBN 2	*Viscosia sp.*	HM564595	HM564802	
SBN 3	*Oxystomina sp.*	HM564596	HM564803	
SBN 4	*Viscosia sp.*	HM564597	HM564804	
SUS 1	*Enoplolaimus sp.*	HM564460	HM564716	HM565002
SUS 2	*Enoplolaimus sp.*	HM564463	HM564719	HM565005

SUS 6	*Enoplolaimus sp.*	HM564466	HM564722	HM565007
SUS 10	*Enoplolaimus sp.*	HM564461	HM564717	HM565003
SUS 15	*Enoplolaimus sp.*	HM564462	HM564718	HM565004
SUS 21	*Enoplolaimus sp.*	HM564464	HM564720	HM565006
SUS 27	Oncholaimidae sp.	HM564465	HM564721	
TCR 1	*Halalaimus sp.*	HM564598	HM564830	
TCR 3	*Halalaimus sp.*	HM564636	HM564809	
TCR 12	Oncholaimidae sp.	HM564605	HM564805	
TCR 13	*Halalaimus sp.*	HM564608	HM564838	
TCR 17	Oncholaimidae sp.	HM564620	HM564806	
TCR 21	*Oxystomina sp.*	HM564631	HM564807	
TCR 26	*Halalaimus sp.*	HM564635	HM564808	
TCR 41	*(Bathyeurystomina sp.)*			HM564977
TCR 42	*Oncholaimidae sp.*	HM564637	HM564862	
TCR 44	*Anticoma sp.*	HM564638	HM564863	
TCR 68	*Oxystomina sp.*	HM564639	HM564864	
TCR 69	Oncholaimidae sp.	HM564640	HM564865	
TCR 70	Phanodermatidae sp.	HM564641	HM564866	HM564978
TCR 74	Thoracostomopsidae sp.	HM564642	HM564867	
TCR 75	Phanodermatidae sp.	HM564643	HM564868	HM564979
TCR 78	*Phanodermopsis sp.*	HM564644	HM564869	HM564980
TCR 80	*Phanodermopsis sp.*	HM564645	HM564870	
TCR 81	*Bathyeurystomina sp.*	HM564646	HM564871	HM564981
TCR 82	Comesomatidae sp.	HM564647	HM564872	
TCR 87	*Bathylaimus sp.*	HM564648	HM564873	HM564982
TCR 89	*Litinium sp.*	HM564649	HM564874	HM564983
TCR 90	*Litinium sp.*	HM564650	HM564875	
TCR 91	*Oxystomina sp.*	HM564651	HM564876	
TCR 93	*Halalaimus sp.*	HM564652	HM564877	
TCR 94	*Mesacanthion/ Paramesacanthi*on sp.	HM564653	HM564878	
TCR 95	*Enoploides sp.*			HM564984
TCR 97	*Cricohalalaimus sp.*	HM564654	HM564879	
TCR 102	Thoracostomopsidae sp.	HM564599	HM564831	
TCR 106	*Bathyeurystomina sp.*	HM564600	HM564832	
TCR 108	*Phanodermopsis sp.*	HM564601	HM564833	
TCR 109	*Bathyeurystomina sp.*	HM564602	HM564834	
TCR 112	*Halalaimus sp.*	HM564603	HM564835	
TCR 114	*Dolicholaimus sp.*	HM564604	HM564836	
TCR 125	*Rhabdocoma sp.*	HM564606	HM564837	
TCR 128	*Bathyeurystomina sp.*	HM564607	HM564910	
TCR 130	*Rhabdocoma sp.*	HM564609	HM564839	
TCR 131	*Halalaimus sp.*	HM564610	HM564840	

TCR 139	*Rhabdocoma sp.*	HM564611	HM564841	
TCR 141	*(Cephalanticoma sp.)*	HM564612	HM564842	
TCR 143	*Enoplolaimus/Mesacanthion sp.*	HM564613	HM564843	
TCR 145	*Syringolaimus sp.*	HM564614	HM564844	
TCR 148	*Phanodermopsis sp.*	HM564615	HM564845	
TCR 149	Anticomidae sp.	HM564616	HM564846	
TCR 152	*Phanodermopsis sp.*	HM564617	HM564847	
TCR 153	Phanodermatidae sp.	HM564618	HM564848	
TCR 158	*Mesacanthion/Paramesacanthion sp.*	HM564619	HM564849	
TCR 173	Phanodermatidae sp.	HM564621	HM564850	
TCR 180	*Oxystomina sp.*	HM564622	HM564851	
TCR 184	*(Epicanthion sp.)*	HM564623	HM564852	
TCR 188	*(Phanodermopsis sp.)*	HM564624	HM564853	
TCR 190	*Phanodermopsis sp.*	HM564625	HM564854	
TCR 192	*Leptosomatides sp.*	HM564626	HM564855	
TCR 197	*Anticoma sp.*	HM564627	HM564856	HM564976
TCR 202	*Oxystomina sp.*	HM564628	HM564857	
TCR 205	*Litinium sp.*	HM564629	HM564858	
TCR 206	*Synonchus sp.*	HM564630	HM564859	
TCR 212	*(Oxystomina sp.)*	HM564632	HM564860	
TCR 216	*(Phanodermopsis sp.)*	HM564633	HM564861	
TCR 230	*Thalassoalaimus sp.*	HM564634	HM564880	
WUS 1	*Enoplolaimus sp.*	HM564467	HM564723	HM565008
WUS 2	*Enoplolaimus sp.*	HM564468	HM564724	HM565009
WUS 3	*Enoplolaimus sp./Mesacanthion sp.*	HM564469	HM564725	
WUS 4	*Enoplolaimus sp.*	HM564470	HM564726	HM565010
WUS 5	*Enoplolaimus sp.*	HM564471	HM564727	HM565011
WUS 6	*Enoplolaimus sp./Mesacanthion sp.*	HM564472	HM564728	
WUS 7	*Enoplolaimus sp.*	HM564473	HM564729	HM565012
