# Supplementary figures and images for: Low endemism, continued deep-shallow interchanges, and evidence for cosmopolitan distributions in free-living marine nematodes (order Enoplida)
Source: BMC Evol Biol. 2010 Dec 18;10:389. doi: 10.1186/1471-2148-10-389 (PMC3022606; doi:10.1186/1471-2148-10-389)

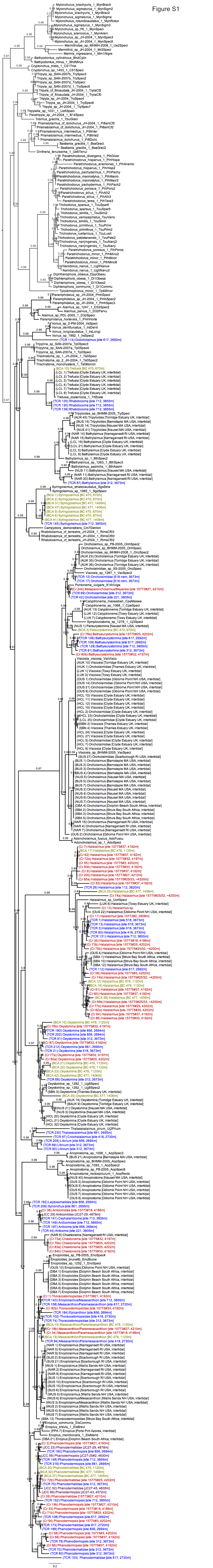

Supplement: Additional file 2 — Expanded Bayesian 18 S Phylogeny of the Enoplida. Figure S1 - Bayesian phylogeny based on SSU data displaying the habitats of marine nematodes within the Enoplida, expanded to show all taxa. Black taxa = shallow-water, red taxa = deep-sea Southern Indian Ocean, blue taxa = deep-sea Pacific, and yellow taxa = Antarctic shelf. Collection depths listed after all deep-sea specimens, and sample codes correspond to sample sites outlined in Table 1. [file 1471-2148-10-389-S2.PNG]
